# Regulation of G Protein-Coupled Receptors by Ubiquitination

**DOI:** 10.3390/ijms18050923

**Published:** 2017-04-27

**Authors:** Kamila Skieterska, Pieter Rondou, Kathleen Van Craenenbroeck

**Affiliations:** 1 Laboratory of GPCR Expression and Signal Transduction (L-GEST), Ghent University, Proeftuinstraat 86, 9000 Ghent, Belgium; k.skieterska@gmail.com (K.S.); Pieter.rondou@ugent.be (P.R.); 2 Center for Medical Genetics Ghent; Ghent University, De Pintelaan 185, 9000 Ghent, Belgium; 3 Cancer Research Institute Ghent (CRIG), Ghent University Hospital, Medical Research Building 2, De Pintelaan 185, 9000 Ghent, Belgium

**Keywords:** G protein-coupled receptors (GPCR), ubiquitination, β-arrestin, deubiquitinating enzyme, E3 ubiquitin ligase

## Abstract

G protein-coupled receptors (GPCRs) comprise the largest family of membrane receptors that control many cellular processes and consequently often serve as drug targets. These receptors undergo a strict regulation by mechanisms such as internalization and desensitization, which are strongly influenced by posttranslational modifications. Ubiquitination is a posttranslational modification with a broad range of functions that is currently gaining increased appreciation as a regulator of GPCR activity. The role of ubiquitination in directing GPCRs for lysosomal degradation has already been well-established. Furthermore, this modification can also play a role in targeting membrane and endoplasmic reticulum-associated receptors to the proteasome. Most recently, ubiquitination was also shown to be involved in GPCR signaling. In this review, we present current knowledge on the molecular basis of GPCR regulation by ubiquitination, and highlight the importance of E3 ubiquitin ligases, deubiquitinating enzymes and β-arrestins. Finally, we discuss classical and newly-discovered functions of ubiquitination in controlling GPCR activity.

## 1. Introduction

### 1.1. GPCR Signaling

G protein-coupled receptors (GPCRs) represent the largest family of membrane proteins that transduce signals from various stimuli, including photons, pheromones, hormones and neurotransmitters. These receptors regulate numerous physiological processes and are the major pharmacological targets in the treatment of many pathological conditions including neurological and cardiovascular disorders, pain, cancer, endocrine and pulmonary diseases [1,2]. Upon activation by their ligands, GPCRs typically signal via heterotrimeric G proteins. However, GPCRs can also signal independently of G proteins, in this situation signaling is mainly mediated by β-arrestins [3,4]. β-arrestins belong to the arrestin protein family which consists of four members: arrestin 1 (visual arrestin), arrestin 2 (β-arrestin 1), arrestin 3 (β-arrestin 2), and arrestin 4 (cone arrestin). The visual and cone arrestin are localized to retina rods and cones and interact mainly with rhodopsin, while the two β-arrestin isoforms are ubiquitously expressed. All four arrestins share high sequence and structural homology [5,6,7]. β-arrestins were first characterized as key regulators of GPCR desensitization and internalization [8,9]; however, it is now known that β-arrestins can regulate GPCR action by functioning as scaffolds for signaling molecules [4,10,11,12,13]. The phenomenon in which specific ligands preferentially activate G protein-or β-arrestin-mediated signaling upon binding to the same receptor is called biased agonism [14].

GPCR signaling is tightly regulated by various mechanisms, including internalization, desensitization, and interaction with cytosolic proteins. These mechanisms are influenced by posttranslational modifications such as phosphorylation, glycosylation, palmitoylation and ubiquitination [15,16,17,18].

### 1.2. Ubiquitination

Ubiquitin is a 76-amino acid polypeptide that is covalently attached to lysine residues in substrate proteins. The attachment of ubiquitin to substrate proteins is processed by a sequential action of three types of enzymes: ubiquitin activating enzymes (E1), ubiquitin conjugating enzymes (E2), and ubiquitin ligases (E3). First, in an adenosine triphosphate (ATP)-dependent process, ubiquitin is activated by E1. In this step, a thiol-ester linkage is formed between the C-terminal glycine of ubiquitin and a cysteine of E1 at the active site. Next, the activated ubiquitin is transferred to a cysteine residue at the active site of E2. Finally, the E3 ligase directly or indirectly catalyzes the covalent attachment of ubiquitin to the target protein. The human genome encodes two E1s, around 60 E2s, and more than 600 E3 ubiquitin ligases; therefore, it is not surprising that E3s are responsible for providing substrate specificity in the ubiquitination process. E3 ligases are mainly categorized into two families: homologous to E6AP C terminus (HECT), and really interesting new gene (RING), based on the structure of the catalytic domains [19]. In mammals, around 30 HECT domain E3s and around 600 RING-type ligases are expressed [19,20]. The HECT ligases possess catalytic activity, they accept ubiquitin from the E2 enzyme and transfer it to the specific residue in the substrate protein [21]; while RING ligases function by bringing E2 enzymes and substrate proteins in close proximity to each other.

Ubiquitin typically binds to the ε-amino group of a lysine residue; however, recent evidence has shown that ubiquitin can also be attached to other residues, including cysteines, serines, threonines, and the N-terminus of the polypeptide backbone [22,23,24,25,26,27,28].

Ubiquitin can be attached to single or multiple residues in the substrate protein representing monoubiquitination and multimonoubiquitination, respectively. Ubiquitin itself contains seven lysine residues (Lys^6^, Lys^11^, Lys^27^, Lys^29^, Lys^33^, Lys^48^, Lys^63^) and they can all potentially be ubiquitinated. Attachment of an additional ubiquitin to a previously substrate-bound ubiquitin molecule leads to polyubiquitin chains of different configurations. Different types of ubiquitination are often associated with different functions. Ubiquitination is a reversible modification and ubiquitin moieties can be removed from the substrate protein by a family of deubiquitinating enzymes (DUBs). The human genome encodes almost 100 DUBs classified into five families: the ubiquitin carboxy-terminal hydrolases, ubiquitin–specific proteases (USPs), ovarian tumor-related proteases, Machado-Joseph disease protein domain proteases, and jab1/MPN domain-associated metalloisopeptidases (JAMM). All families are cysteine proteases, except JAMM, which are a family of metalloproteases [29]. USPs represent the largest family of DUBs (58 members) and most of the described DUBs involved in the regulation of GPCR ubiquitination belong to this family [29,30,31]. We have compiled an overview of all published studies on GPCR ubiquitination (including E3 ubiquitin ligases), DUBs, receptor residues which are ubiquitinated, the role of the ubiquitination, and whether the modification is induced by receptor stimulation (Table 1). In this review, we elaborate on some crucial reports and discuss the role of ubiquitination in the regulation of GPCR function.

## 2. Functional Role of GPCR Ubiquitination

**Figure 1:**
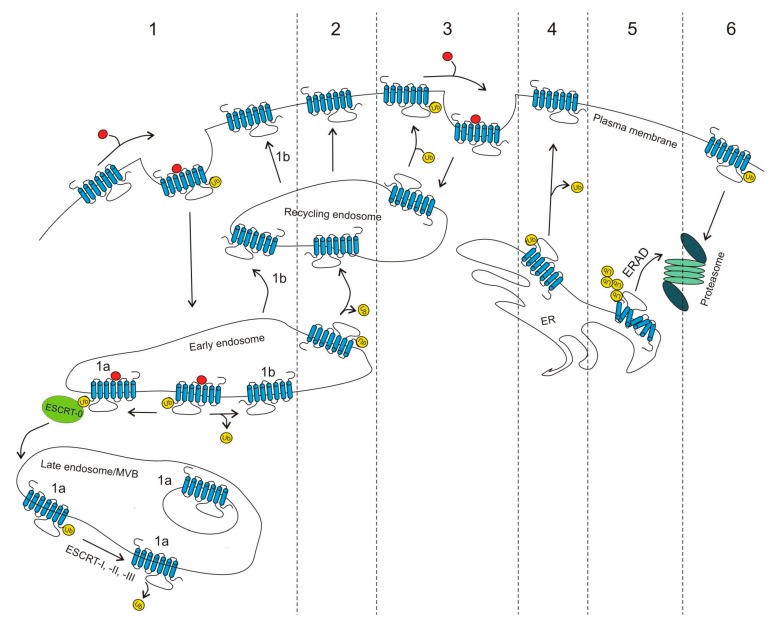
Role of ubiquitin in GPCR trafficking. (**1**) Many GPCRs have been described to undergo agonist-induced ubiquitination and down-regulation. Upon endocytosis, they are often directed for lysosomal degradation via the conserved endosomal-sorting complex required for the transport (ESCRT) machinery (**1a**); however, some of them can be deubiquitinated and directed to the resensitization pathway (**1b**). (**2**) Constitutive ubiquitination of frizzled-4 receptor (FZD_4_R) promotes its internalization and lysosomal degradation, while deubiquitination leads to its recycling and increased cell surface expression. (**3**) Some GPCRs are basally ubiquitinated (steady-state) and upon agonist binding are deubiquitinated and internalized. After subsequent ubiquitination, they can recycle back to the cell surface. (**4**) Some properly folded GPCRs (e.g., A_2A_R) require deubiquitination to be delivered to the cell surface. (**5**) Ubiquitination also functions as a quality control system in which misfolded, polyubiquitinated receptors are directed for proteasomal degradation via the endoplasmic reticulum-associated degradation (ERAD) pathway. (**6**) Some GPCRs are ubiquitinated at the plasma membrane and are directed to the proteasome via a poorly understood process.

Although an increasing number of in-depth studies about ubiquitination of GPCRs are becoming available, our understanding of the function of this modification in the regulation of these receptors is still very limited. The best characterized role of ubiquitination is the targeting of activated GPCRs for lysosomal degradation [87,88,89]. Additionally, ubiquitin has been shown to serve as a tag in the quality control system, the endoplasmic reticulum-associated degradation (ERAD) pathway, and to direct misfolded proteins for proteasomal degradation. Ubiquitination also regulates cell surface expression of GPCRs via different mechanisms and in this way controls cell responsiveness to different stimuli (Figure 1). Recently, the role of ubiquitin in the regulation of GPCR signaling and its involvement in differential cellular responses upon GPCR activation with biased agonists has been demonstrated. These well-defined and novel roles of ubiquitin in GPCR regulation will all be discussed in this review.

### 2.1. Regulation of GPCR Cell-Surface Expression by Ubiquitination

In addition to the transcription, translation, and targeting of the receptor to the plasma membrane, down-regulation plays an important role in the regulation of GPCR expression. There are two major pathways involved in the degradation of GPCRs: the ubiquitin proteasome system (UPS), and the endosomal-sorting complex required for the transport (ESCRT) pathway leading to lysosomal degradation. Additionally, deubiquitinating enzymes and β-arrestins play an important role in the regulation of GPCR trafficking. In this chapter, our focus is on single mechanistic studies, but the entire picture is much more dynamic and diverse. Furthermore, very limited studies have focused on the kinetics of these processes, that is the kinetics of ubiquitination, deubiquitination, and the interaction of proteins involved in the regulation of E3 ligases, DUBs, and proteins important for receptor internalization, etc. Extensive knowledge about these processes is of paramount importance for full understanding of the regulation of GPCR cell-surface expression.

#### 2.1.1. Proteasomal Degradation

The initial discovery of the role of ubiquitin was to serve as a tag to mark misfolded or unneeded proteins for degradation in the proteasome. Ubiquitination of GPCRs was shown to be involved in the ERAD, which mainly serves as a quality control system where misfolded receptors are ubiquitinated, and targeted for degradation in the proteasome [90] (Figure 1, pathway 5). During biogenesis, GPCRs and other transmembrane proteins are folded in the endoplasmic reticulum (ER) and adopt specific conformations. This process is assisted by resident molecular chaperons and folding factors. Next, proteins are released from the ER and are transported through the secretory pathway to the Golgi, and upon complete maturation move to their final destination. Misfolded proteins are retrotranslocated from the ER to the cytosol and upon ubiquitination are further directed to the proteasome for proteolytic degradation [64,91]. The 26S proteasome is a protein complex consisting of a 20S protein subunit with a proteolytic function and two 19S regulatory cap subunits. Proteins that are tagged by polyubiquitin chains consisting of at least four ubiquitin moieties are recognized by 19S regulatory subunits. Ubiquitin is then cleaved and recycled while targeted proteins are degraded in the 20S subunit. Lys^48^-type polyubiquitin chains serve most frequently as signals for proteasomal degradation, but other linkages such as Lys^63^-type, Lys^11^-type, linear and non-lysine ubiquitination chains can also be recognized by the proteasome [92,93,94,95].

In this way, only properly folded receptors are delivered to the membrane. The importance of ERAD as a quality control system during biogenesis was shown for the calcium-sensing receptor [83], thyrotropin-releasing hormone receptor [79], μ-opioid receptor (MOR), δ-opioid receptor (DOR) [64,68], and dopamine D_4_ receptor (D_4_R) [96].

However, there have been examples of properly folded GPCRs that are deubiquitinated during biosynthesis and transported to the cell surface, for example, the adenosine receptor A_2A_ [32]. Furthermore, several GPCRs have been described to undergo basal and agonist-induced proteasomal degradation (Figure 1, pathway 6). Metabotropic glutamate receptors 1 and 5 (mGluR_1_ and mGluR_5_) and the human follitropin receptor are directed to the proteasome, which does not depend on agonist stimulation [54,85]. Additionally, proteasomal degradation has been shown to play an important role in the regulation of different opioid receptors. MOR and DOR agonist-induced down-regulation were attenuated upon pretreatment of the cells with proteasome inhibitors (Z-Leu-Leu-Leu-al, ZLLL; lactacystin; Z-Ile-Glu(OtBu), PSI and Ac-Leu-Leu-nLeu-al, ALLN), while pretreatment with a lysosome inhibitor (E64d) had little effect. In addition, incubation with all tested proteasome inhibitors in the absence of an agonist increased the steady state MOR and DOR levels, indicating that proteasomal degradation also has an important role in the basal turn-over of both receptors [68]. The κ-opioid receptor (KOR), ubiquitinated after phosphorylation and Lys^63^-type polyubiquitination, was shown as a dominant form of KOR ubiquitination. Agonist-induced ubiquitination of KOR leads to its down-regulation, but surprisingly, the lysine-deficient mutant with a clear decrease in ubiquitination was also down-regulated, suggesting that more than one mechanism may control receptor degradation [66]. Additionally, a combination of both proteasome and lysosome inhibitors was required to completely block KOR degradation, suggesting that both pathways are involved in agonist-promoted down-regulation of the receptor [65]. Although the effect of proteasome inhibitors was observed in the above-mentioned examples, it is not clear how mature receptors can be extracted from the cell membrane and directed to the proteasome. It is possible that the proteasome indirectly influences receptor degradation, that is via proteins involved in the degradative pathway. Such a scenario has been previously proposed by Hicke [97].

#### 2.1.2. Lysosomal Degradation

GPCRs that recognize and integrate signals from various stimuli need to undergo strict regulation to prevent cell overstimulation. One of the main regulatory mechanisms is receptor desensitization, followed by receptor internalization, and subsequent degradation of the receptor in the lysosomes (Figure 1, pathway 1).

The involvement of ubiquitin in the regulation of GPCR trafficking was first demonstrated in 1996 in yeast, and showed that ubiquitination was necessary for the internalization and degradation of the sterile 2 α-factor receptor protein (Ste2p) [98,99]. In mammals, however, ubiquitination is not required for receptor internalization, but seems to regulate, via different mechanisms, lysosomal degradation of the activated receptor via the highly conserved endosomal-sorting complex required for transport (ESCRT) pathway [100,101]. The ESCRT machinery consists of four distinct protein complexes known as ESCRT-0, -I, -II and -III. ESCRT complexes (together with numerous accessory proteins) act sequentially to sort ubiquitinated cargo from early endosomes into intraluminal vesicles (ILVs) of multivesicular bodies (MVBs) before degradation in the lysosome [102].

Ubiquitin-dependent sorting of GPCRs via ESCRT to lysosomes has been shown for many GPCRs (Table 1). The C-X-C chemokine receptor-4 (CXCR4) and proteinase-activated receptor 2 (PAR2) are ubiquitinated and sorted for lysosomal degradation upon agonist stimulation. Alterations in proper ubiquitination of these receptors prevent their lysosomal sorting and degradation. Moreover, disruption of ESCRT components also blocked CXCR4 and PAR2 sorting to lysosomes, confirming the importance of this pathway in receptor degradation [46,47,77,103,104]. Other examples of GPCRs where ubiquitination has been shown to play a role in lysosomal sorting after prolonged agonist treatment are the neurokinin-1 receptor (NK_1_R) and MOR. When ubiquitination is impaired upon internalization, NK_1_R is recycled back into the plasma membrane [78]. MOR ubiquitination is necessary for its ESCRT-dependent down-regulation and controls receptor distribution between the limiting endosome membrane and lumen, but is not essential for receptor delivery to the proteolytic compartments. Instead, this is dictated by the MOR C-terminal tail and is independent of receptor ubiquitination [69].

The prototypic β_2_-adrenergic receptor (β_2_AR) was shown to be sorted to late endosomes/lysosomes upon agonist (isoproterenol) stimulation, while the mutant receptor (lacking lysines (β_2_AR-K0)) was not ubiquitinated and showed resistance to lysosomal degradation [34,37]. Treatment of the β_2_AR with β-arrestin biased agonist (carvedilol) also resulted in ubiquitination and lysosomal sorting of the receptor. This appears to be a distinct type of ubiquitination, rather than the one induced by a balanced agonist in which another E3 ligase is involved and where ubiquitin is probably attached to other non-lysine residues [38]. Upon balanced agonist (isoproterenol) treatment, β_2_AR is ubiquitinated by the HECT-type E3 ligase neural precursor cell-expressed developmentally downregulated gene 4 (Nedd4) [36]; while upon β-arrestin biased agonist treatment (carvedilol), the RING-type ubiquitin ligase membrane associated ring-CH-type finger 2 (MARCH2) ubiquitinates the wild type receptor as well as the lysine-lacking mutant [38].

Although ubiquitination plays an important role in GPCR lysosomal degradation, there are examples of receptors which can efficiently sort to lysosomes in a ubiquitin-independent manner. The calcitonin-like receptor (which is not ubiquitinated upon activation) and a lysine-deficient mutant of DOR (of which ubiquitination is completely blocked) are still degraded in the lysosomes. These receptors require an ESCRT-0 complex component for their lysosome sorting, confirming that ubiquitination is not obligatory for GPCRs to enter the ESCRT pathway [60,62,103,105]. In contrast, proteinase-activated receptor 1 (PAR1) undergoes agonist-induced ubiquitination and is directed to lysosomes in the next step. However, its degradation is independent of receptor ubiquitination and ubiquitin-binding components of ESCRT-0 and ESCRT-I complexes *(*hepatocyte growth factor-regulated tyrosine kinase substrate—HRS and Tsg101) [75,106,107]. A later study revealed that ALG-2-interacting protein X (ALIX), an ESCRT-III interacting protein, interacts with the YPX_3_L motif in the second intracellular loop of PAR1 and purinergic P2Y_1_ receptor, and is necessary for receptor lysosomal sorting. This conserved motif was also found in the second intracellular loop of several other GPCRs, suggesting that regulation of lysosomal sorting by ALIX could be a common mechanism within the GPCR family [61,74,108,109]. Most recently, it was shown that ALIX is regulated by the arrestin domain-containing protein 3 (ARRDC3), and this regulation is necessary for ALIX to sort PAR1 to the lysosomes. Upon PAR1 activation, ARRDC3 recruits HECT-type E3 ubiquitin ligase WW domain-containing protein 2 (WWP2) which ubiquitinates ALIX. In this way, ARRDC3 facilitates the sorting of PAR1 into ILVs of MVBs [110]. The mechanisms that control ALIX-dependent GPCR sorting at the multivesicular endosomes remain poorly understood; however, it is speculated that ubiquitin may regulate ALIX by facilitating its dimerization, which in turn can promote its interaction with the ESCRT-III complex [110].

This finding demonstrates that direct ubiquitination of the GPCR might not be obligatory for its degradation, but that ubiquitin may still play an important regulatory role in GPCR lysosomal sorting by modifying other proteins involved in this process.

#### 2.1.3. Deubiquitination and GPCR Cell-Surface Expression

Ubiquitination can direct receptors for degradation while also regulating the cell-surface expression of GPCRs. The latter involves the dynamic actions of ubiquitin ligases and DUBs, and in this way modulates cell responsiveness [111].

GPCRs that undergo agonist-induced ubiquitination are usually internalized and targeted for degradation in lysosomes. However, after deubiquitination they can often be redirected to the resensitization pathway and recycled back to the cell surface (Figure 1, pathway 2). Upon stimulation with the agonist isoproterenol, β_2_AR is internalized and undergoes lysosomal degradation, but its deubiquitination by USP33 and USP20 on late endosomes promotes receptor recycling to the plasma membrane [33]. These USPs are coupled to the receptor in a non-active state and upon agonist stimulation are transferred to β-arrestin 2. This leads to deubiquitination of β-arrestin 2 and its dissociation from the receptor upon internalization [33]. At the late endosomes, USPs can reassociate with the receptor and allow its recycling to the cell surface. It has also been shown that the presence of only one USP is sufficient for receptor recycling. Most recently, it was described that upon physiological stress, USP20 is phosphorylated by protein kinase A and this process regulates trafficking of β_2_AR to autophagosomes [112].

Ubiquitination of CXCR4 by HECT-type E3 ligase atrophin-1-interacting protein 4 (AIP4) induced by C-X-C motif chemokine ligand 12 (CXCL12) leads to receptor lysosomal degradation [113]. Overexpression of USP14 promotes CXCR4 deubiquitination and allows it to escape degradation [42,48]. Another interesting example represents frizzled-4 receptor (FZD_4_R) where a balance between ubiquitination and deubiquitination is important for regulating its membrane expression. Constitutive ubiquitination of FZD_4_R promotes its internalization and lysosomal degradation, while deubiquitination mediated by USP8 leads to recycling and increased cell surface expression [86]. USP8 has also been shown together with another DUB, associated molecule with the SH3-domain of STAM (AMSH), to regulate PAR2 trafficking. These two DUBs are associated with the ESCRT pathway. USP8 and AMSH mediate deubiquitination of PAR2 and its sorting from endosomes to lysosomes [76]. AMSH and USP8 may also regulate trafficking of DOR, but it is unclear whether they do so through direct deubiquitination of DOR, or via regulation of the ESCRT machinery [61]. Furthermore, the importance of these two DUBs has also been shown in the regulation of CXCR4 ubiquitination; however, in this case, they do not work through direct deubiquitination of the receptor. USP8 regulates CXCR4 lysosomal degradation, but does so through the deubiquitination of the ESCRT machinery and not CXCR4 [41]. AMSH was shown to not play a role in the agonist-induced lysosomal degradation of CXCR4, in contrast to its role in PAR2 regulation [44,114]. Additionally, deubiquitination and lysosomal degradation of CXCR4 is regulated directly by USP14 [48]. Overexpression of this DUB has been shown to prevent lysosomal degradation of CXCR4 [42]. These examples highlight that the ubiquitination of a single receptor can be regulated by several DUBs each working with a different mechanism, and that the same DUB can change its mechanism of action depending on the receptor which undergoes regulation.

Additionally, there are GPCRs which require deubiquitination to enter the internalization pathway (Figure 1, pathway 3). The C-X-C chemokine receptor-7 (CXCR7) is ubiquitinated in a basal state on a lysine residue located in the C-terminus. Receptor activation with CXCL12 leads to its reversible deubiquitination in a process that requires phosphorylation and β-arrestin recruitment [49]. When the ligand is removed, the receptor is ubiquitinated again and recycled back to the plasma membrane.

Like CXCR7, PAR1 was described as constitutively ubiquitinated. Agonist stimulation of PAR1 induces its deubiquitination [75]; however, after its internalization, ubiquitination of PAR1 increases once again [74]. Lysine-less PAR1 mutants displayed constitutive endocytosis, suggesting that ubiquitination is necessary for membrane expression of the receptor and negatively regulates its constitutive internalization [75].

In contrast to these observations, the deubiquitination of the adenosine A_2A_ receptor (A_2A_R) by USP4 is necessary for the cell surface delivery of a functionally active receptor (Figure 1, pathway 4) [32].

The parathyroid hormone 1 receptor (PTH1R) regulates bone growth and extracellular mineral ion homeostasis and represents another interesting example of a GPCR regulated by integrated ubiquitination and deubiquitination mechanisms. This receptor responds to distinct ligands, and stimulation with both the activating ligand PTH (1–34) and the non-activating ligand PTH (7–34) leads to polyubiquitination of PTH1R. The enzymes involved in PTH1R ubiquitination are still unknown, although an enzyme (USP2) involved in deubiquitination of the receptor has been described. Upregulation of USP2 mRNA upon treatment with the activating ligands has been shown in bone [115], and was also detected in rat osteosarcoma cells [82]. The different regulation of USP2 can explain why ubiquitination induced by the activating ligand PTH (1–34) is transient and the non-activating ligand PTH (7–34) is sustained. This difference in receptor ubiquitination has important implications as PTH1R undergoes fast deubiquitination and recycles to the cell membrane upon treatment with the activating ligand PTH (1–34). In contrast, the non-activating ligand PTH (7–34) induces sustained ubiquitination of PTH1R which leads to its proteasomal degradation [82].

#### 2.1.4. β-Arrestins, Ubiquitination and GPCR Trafficking

Our knowledge on the role of β-arrestins in GPCR regulation has increased rapidly. From regulation of GPCR internalization and desensitization, to mediating G protein-independent signaling, β-arrestins appear to be crucial components of the complicated protein network involved in controlling proper GPCR function. β-arrestins function as adaptors for E3 ubiquitin ligases [116,117] or deubiquitinating enzymes [41,118] and as such they play an important role in the regulation of GPCR ubiquitination. However, at times, their mechanism of action is not well defined. The first research on the importance of β-arrestins in the regulation of GPCRs by ubiquitination came from the studies of Shenoy et al. [34] who demonstrated that agonist-induced activation of the β_2_AR led to the transient ubiquitination of the receptor and of β-arrestin 2. Next, they showed that the mouse double minute 2 homolog (MDM2) ligase was important for the ubiquitination of β-arrestin 2, but that MDM2 depletion had no effect on receptor ubiquitination. Later, it was revealed that another E3 ligase, Nedd4, was responsible for the ubiquitination of β_2_AR, and that β-arrestin 2 functioned as an adaptor to recruit Nedd4 to activated β_2_AR [36]. A recent study showed that Nedd4 may be recruited to β_2_AR independently of β-arrestin 2 through a mechanism mediated by ARRDC3, a member of the α-arrestin protein family [119]. Nabhan et al. [119] showed that ARRDC3 interacted with Nedd4 and β_2_AR and served as an adaptor for ubiquitination of the receptor mediated by Nedd4. Knockdown of ARRDC3 abolished agonist-induced ubiquitination and lysosomal degradation of β_2_AR. However, another group [120] recently presented a report that β-arrestin 2, and not ARRDC3, was the adaptor protein necessary for the ubiquitination of β_2_AR. They also concluded that β-arrestin 2 and ARRDC3 functioned sequentially. Upon β_2_AR activation, β-arrestin 2 mediated the ubiquitination of the receptor by recruiting Nedd4 and promoted receptor endocytosis. ARRDC3 (as well as other ARRDC proteins (2 and 4)) functioned as secondary adaptors recruited to internalized β_2_AR-Nedd4 complexes on endosomes [120]. β-arrestin 1 was shown to bind and co-localize with HECT-type AIP4 ubiquitin ligases on early endosomes upon CXCR4 activation [42]. Depletion of β-arrestin 1 blocked agonist promoted degradation of CXCR4 by preventing its trafficking from early endosomes to lysosomes. Interestingly, ubiquitination and internalization of CXCR4 were not affected, suggesting that the interaction between β-arrestin 1 and AIP4 was not required for ubiquitination of the receptor at the plasma membrane, but was probably important for subsequent post-internalization events. One possible explanation is the adaptor function of β-arrestin 1 for AIP4-mediated ubiquitination of ESCRT-0 components. β-arrestin 1 was shown to interact with signal transducing adapter molecule 1 (STAM-1) (a subunit of ESCRT-0) [44]. Disruption of this interaction reduced ubiquitination of the second ESCRT-0 subunit, HRS, which in turn enhanced CXCR4 degradation. Involvement of β-arrestins in the regulation of ubiquitination of other GPCRs (such as the MOR and vasopressin V_2_ receptor (V_2_R)) was also demonstrated, but the precise role of β-arrestins in these processes was unclear. Using β-arrestin1/2 knockout mouse embryonic fibroblast (MEF) cells, Groer et al. [67] showed that β-arrestin1 was crucial for [d-Ala^2^, *N*-MePhe^4^, Gly-ol]-enkephalin (DAMGO) mediated ubiquitination while morphine (the other agonist) could not induce MOR ubiquitination, nor when G protein-coupled receptor kinase 2 (GRK2) was overexpressed. As the scaffolding machinery has not been investigated, we do not know which E3 ligase is involved. However, another study [59] showed that stimulation of MOR with the non-selective agonist [D-Ala^2^, D-Leu^5^]-Enkephalin (DADLE) induced ubiquitination of MOR by the HECT-type E3 ligase Smurf2.

In case of V_2_R, β-arrestin 2 was shown to be the critical component in the rapid ubiquitination and degradation of the receptor upon agonist stimulation [80].

Although β-arrestins are mainly adaptors for E3 ubiquitin ligases, one study [49] showed that constitutively ubiquitinated CXCR7, upon stimulation, underwent deubiquitination in a process that required phosphorylation and β-arrestin recruitment. The authors proposed a model in which β-arrestins may function as scaffolds for deubiquitinating enzymes, but the exact function of these proteins in the regulation of CXCR7 ubiquitination still needs to be investigated.

All the studies presented clearly show that β-arrestins are very important regulators of GPCR ubiquitination, although their precise role in this process is still poorly understood.

### 2.2. Importance of Ubiquitination in GPCR Signaling and Biased Agonism 

#### 2.2.1. GPCR Signaling

Although most studies have highlighted the role of ubiquitination as a mechanism controlling GPCR down-regulation, there is also evidence that ubiquitin influences GPCR signaling. Many GPCRs signal via activation of the mitogen-activated protein kinase (MAPK) pathway, also known as the extracellular-signal regulated kinase (ERK) pathway. The MAPK pathway is a chain of proteins in a cell that transfer the signal from the receptor on the cell surface to the DNA in the nucleus, or to other subcellular targets causing cellular responses including proliferation, gene expression, differentiation, mitosis, cell survival, and apoptosis. Signaling molecules in this pathway communicate between each other by adding a phosphate group to the neighboring proteins. This phosphorylation event functions as an “on/off” signal, leading to the activation or inhibition of the next signaling molecule in the chain [121]. The most commonly studied MAPK pathway initiated by GPCR activation is the p44/42 MAPK (ERK1/2) pathway. This pathway can be activated by G protein-dependent and -independent mechanisms [122,123,124]. The G protein-independent MAPK signaling often requires β-arrestins as scaffolds for the signaling complexes [10,125,126]. We can assume that the scaffold function of β-arrestins is related to their ubiquitination status and their ability to co-internalize with certain GPCRs. The ubiquitination of β-arrestin 2 is controlled by the ubiquitin ligase Mdm2 and the deubiquitinating enzyme USP33, and is regulated by the activation of GPCRs, e.g., β_2_AR [35]. Based on the affinity of β-arrestin and trafficking patterns, GPCRs are divided into two classes. Receptors from class A (e.g., β_2_AR) form a transient complex with β-arrestin 2 and internalize without them, which promotes fast recycling. In contrast, receptors from class B (e.g., V_2_R) form more stable complexes with β-arrestin, internalize into endosomes together with β-arrestin 2 and are slowly recycled. Stimulation of the β_2_AR and V_2_R leads to transient or stable β-arrestin ubiquitination, respectively. Thus, ubiquitinated β-arrestin internalizes (together with the receptor) towards endosomes with activated MAPK. Such a situation was demonstrated for AT_1a_R [127,128]. The precise mechanism demonstrating how ubiquitination of β-arrestin 2 favors sustained p44/42 MAPK activation is unclear. It is possible that β-arrestin 2-bound ubiquitin allows a more stable interaction between β-arrestin 2 and the receptor which promotes their co-internalization and sustained MAPK activation on endosomes. Furthermore, ubiquitinated β-arrestin 2 may work as an adaptor for ubiquitin binding domain (UBD) proteins which mediate sustained MAPK signaling [129].

Another study indicated that ubiquitination may play a role in the regulation of G protein-dependent signaling. CXCR4-mediated p44/42 MAPK phosphorylation is G protein-dependent and does not require β-arrestin. Malik et al. [45] showed that two proteins crucial for lysosomal sorting of CXCR4 (the ubiquitin E3 ligase AIP4 and the ESCRT-0 subunit STAM-1) were also involved in the regulation of CXCR4-mediated p44/42 MAPK activation. Depletion of either AIP4 or STAM-1, as well as inhibition of the interaction between these two proteins, blocked phosphorylation of p44/42 MAPK upon CXCR4 activation. Moreover, it was observed that there was a discrete subpopulation of AIP4 and STAM-1 in caveolar microdomains with CXCR4 which appeared to mediate MAPK activation. To explain this observation, the authors proposed a mechanism in which ubiquitination of STAM-1 by AIP4 in caveolae regulated the activation of p44/42 MAPK signaling initiated by CXCR4, but further research is required for clarification [45]. Interestingly, extracellular ubiquitin can bind to the CXCR4 receptor and functions as a natural agonist of this receptor. Binding of the extracellular ubiquitin promotes intracellular Ca^2+^ flux and reduces cAMP levels via the Gαi/o protein [130]. These studies present a novel idea on the importance of ubiquitin in the regulation of GPCR signaling. Ubiquitin does not only influence receptor signaling by modifying the receptor itself or various receptor interacting proteins, but can also serve as a direct activator of the receptor on the extracellular site of the cell.

Recently, signaling via another chemokine receptor, CXCR2, was shown to be strictly dependent on the ubiquitination status of the receptor. A single lysine residue (Lys^327^) in the C-terminal tail of CXCR2 was identified as a site of ubiquitination. Moreover, this ubiquitination seemed to play a key role in receptor internalization and signaling. CXCR2, where Lys^327^ was mutated to arginine, displayed decreased polyubiquitination, failed to recruit β-arrestin, and did not internalize upon stimulation of the receptor. Furthermore, the intracellular signaling—including both early events, such as MAPK phosphorylation and the increase in calcium flux, and the later activation of the nuclear factor kappa-light-chain-enhancer of activated B cells (NF-κB) and activator protein 1 (AP1) transcription factors—was blunted. The precise mechanism of how the ubiquitination of a single lysine can regulate so many aspects of CXCR2 signaling still needs to be elucidated [40]. In addition, a study where all lysines in the C-terminal tail of the CXCR2 were mutated [131] showed that these residues were not crucial for CXCR2 degradation in the lysosomes.

Ubiquitination is also important for p38 MAPK activation. It was revealed that activation of PAR1 with α-thrombin led to its Lys^63^-type polyubiquitination mediated by the Nedd4-2 E3 ubiquitin ligase [71]. Ubiquitinated PAR1 recruits the transforming growth factor-β-activated protein kinase-1 binding protein-2 (TAB2) which binds to the receptor via its ubiquitin binding motif and functions as an adaptor for TAB1 protein, resulting in TAB2-TAB1-p38 signaling complex formation on endosomes. The phosphorylation of p38 occurred in a non-canonical way as the two kinases typically necessary for this process, mitogen-activated protein kinase kinase 3 (MKK3) and MKK6, were not obligatory in the case of PAR1 initiated p38 phosphorylation. Ubiquitin and TAB-dependent activation of p38 was required for thrombin-induced endothelial barrier permeability in vitro, and signaling by p38 MAPK was essential for PAR1-stimulated vascular leakage in vivo. Interestingly, ubiquitination of the purinergic GPCR P2Y was also required for the TAB-dependent activation of p38 MAPK upon receptor activation [71]. This finding suggests that non-canonical p38 signaling is not limited to PAR1 and can be a more general phenomenon with an important role in GPCR ubiquitination.

#### 2.2.2. Biased Agonism

Biased agonism is a phenomenon where different ligands elicit distinct responses at the same receptor [14]. Understanding the molecular basis of biased agonism is of great importance in pharmacology as it will allow the development of drugs which produce beneficial responses with limited side effects [3,132,133,134]. Recent studies have demonstrated that ubiquitination is important in determining differential GPCR regulation induced by biased agonists. Here we provide examples that show that various ligands induce distinct ubiquitination patterns of the same receptor.

MOR, a major target for opiate drugs, is a receptor that displays various responses when activated with different ligands [135]. Morphine and DAMGO (two MOR-specific agonists) cause a difference in the recruitment of β-arrestins (1/2) and in MOR ubiquitination. Activation of MOR with DAMGO leads to MOR internalization mediated by β-arrestin 1 and β-arrestin 2. In contrast, morphine induces only β-arrestin 2 recruitment, followed by receptor internalization [67]. Next, there was a huge difference in the ubiquitination of MOR upon incubation with morphine or DAMGO (as DAMGO induces MOR ubiquitination), while morphine treatment had no effect on the ubiquitination of the receptor. Further experiments proved that β-arrestin 1 (and not β-arrestin 2) was important in MOR ubiquitination. Another study [62] showed that stimulation of MOR with the non-selective agonist DADLE also induced ubiquitination of the receptor, which controlled its internalization as ubiquitin-deficient MOR did not internalize through clathrin-coated pits as efficiently as wild-type receptors.

Another interesting example (previously mentioned in Section 2.1.2) is β_2_AR, which can be ubiquitinated by different E3 ligases upon treatment with different ligands. Upon activation of β_2_AR with its balanced agonist isoproterenol, the receptor undergoes rapid ubiquitination by the HECT-type ubiquitin ligase Nedd4, followed by lysosomal degradation [36]. The β-arrestin biased agonist carvedilol stimulates β-arrestin2-dependent signaling, but is also an antagonist for G protein-dependent signalling [136]. Stimulation of β_2_AR with carvedilol also leads to ubiquitination and lysosomal degradation, but ubiquitination is now added to the receptor via the RING-type E3 ligase MARCH2 [38].

All examples presented above show that distinct ligands can differentially modulate the activity of the ubiquitin machinery that plays a role in the regulation of biased signaling.

### 2.3. Transubiquitination

GPCRs are known to transactivate other receptors such as growth factor tyrosine kinase receptors via release of membrane-anchored ligands, or through the modulation of receptor cytoplasmic domains [137]. Recent studies have revealed that some receptors and antagonists can stimulate GPCR transubiquitination and degradation. The orexin receptor (OX_2_), involved in the regulation of the sleep/wake cycle, is ubiquitinated and degraded in response to signaling by the proinflammatory cytokine tumor necrosis factor α (TNF-α) [70]. An example of an antagonist which promotes ubiquitination of a GPCR is FTY720. This inhibitor of sphingosine-1-phosphate receptor (S1PR) signaling induces phosphorylation of the C-terminal domain of the receptor followed by receptor internalization, polyubiquitination, and proteasomal degradation, and finally results in a functional antagonism of S1P signaling [56,57].

**Figure 2:**
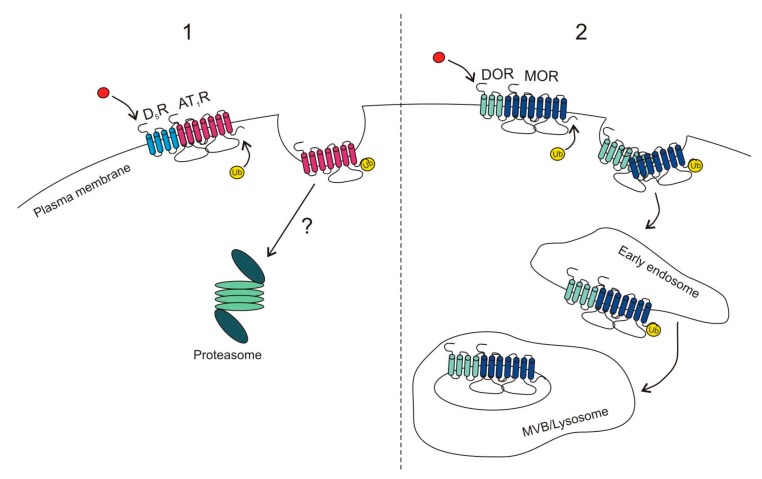
Transubiquitination between GPCRs: Activation of one GPCR can promote ubiquitination of another GPCR. (**1**) Agonist stimulation of dopamine D_5_ receptor (D_5_R) leads to the ubiquitination of angiotensin-II type 1 receptor (AT_1_R), disruption of the interaction between the two receptors, and subsequent proteasomal degradation of AT_1_R. The precise mechanism is poorly understood; (**2**) Activation of δ-opioid receptor (DOR) with its specific agonist leads to the ubiquitination of μ-opioid receptor (MOR) and co-internalization of both receptors which are in the next steps targeted for lysosomal degradation. This causes a decrease in cell responsiveness to opiate agonists.

Moreover, certain GPCRs promote transubiquitination of other GPCRs which can dramatically change cell responsiveness. This phenomenon was first described for the angiotensin-II type 1 receptor (AT_1_R) in response to dopamine D_5_ receptor (D_5_R) activation. Both receptors have opposite influences on cellular signal transduction, where signaling of AT_1_R is prohypertensive while D_5_R signaling is antihypertensive. Li et al. [39] demonstrated that activation of D_5_R promoted ubiquitination and proteasomal degradation of AT_1_R, but it was unclear whether the two receptors interacted directly with each other, or if it was an indirect effect mediated by downstream signaling molecules (Figure 2(1)). 

Accumulating evidence suggests that GPCRs can also form functional homo- and heteromers as well as higher order oligomers which exhibit different signaling properties when compared to monomers [138,139,140,141,142,143,144,145,146,147]. DOR and MOR were shown to form heteromers, and depending on the ligand used, the heteromers were recycled or directed for lysosomal degradation [63]. Stimulation of DOR with its specific agonist (Delt I) promoted ubiquitination, but not phosphorylation of MOR and led to degradation of both receptors (Figure 2(2)). Disruption of the heteromer by an interfering peptide containing the first transmembrane domain of MOR rescued MOR cell-surface expression and increased cell sensitivity to opiate agonists [63], while DOR could still undergo internalization.

## 3. Conclusions

It is widely accepted that ubiquitination plays a vital role in the regulation of many cellular processes. However, our knowledge about the exact functions of ubiquitination in the coordination of GPCR action is still limited. The increasing amount of research in this field highlights the huge importance of E3 ubiquitin ligases, DUBs and other GPCR-interacting proteins (like β-arrestins) in coordinating complicated ubiquitin-mediated processes in a very flexible way. The role of ubiquitination in regulating lysosomal degradation of activated GPCRs is already well-established. Recent findings have provided evidence for the additional involvement of ubiquitin in many other regulatory mechanisms like receptor trafficking, β-arrestin-, and G protein-mediated signaling, and even in the modulation of differential responses initiated by biased agonists. The incredible diversity of the mechanisms directly or indirectly controlled by ubiquitination warrants future studies to uncover additional, yet unknown functions of this posttranslational modification in GPCR regulation. Amongst others, these studies should focus on identifying proteins which are involved in or are targets of ubiquitination initiated by the activation of specific GPCRs. Finally, understanding the molecular basis of the regulatory processes controlled by ubiquitin will be of great value in the development of new drugs and therapies.

## Figures and Tables

**Table 1:** G protein-coupled receptors (GPCR) ubiquitination. The table presents a summary of current knowledge regarding GPCR ubiquitination. The list includes the GPCR family, receptor name and available information on receptor ubiquitination, including E3 ligase, and deubiquitinating enzymes (DUB) and ubiquitin-binding sites. Next, we mention whether ubiquitination is agonist induced or constitutive. Finally, the reported effect on GPCR function is stated.

GPCR	E3 Ligase	DUB	Residues	Induced/Constitutive	Role	Comment	Reference
Class A GPCRs							
Adenosine receptors							
A_2A_	N.D.	USP4	N.D.	Constitutive	N.D.	Deubiquitination necessary for surface expression	[32]
Adrenoceptors							
β2	Nedd4	USP20, USP33	Lys in IC3 and C-term.	Agonist (isoproterenol)	Lysosomal degradation, regulation of arrestin-mediated signaling	β-arrestin 2 involved	[33,34,35,36,37]
	MARCH2	N.D.	Non-Lys	β-arrestin biased agonist (carvedilol)	Lysosomal degradation	N.D.	[38]
Angiotensin receptors							
AT_1_	N.D.	N.D.	N.D.	Activation of D_5_R (Fenoldopam)	Proteasomal degradation of glycosylated receptor	Polyubiquitination	[39]
Chemokine receptors							
CXCR2	N.D.	N.D.	Lys^327^	Agonist (IL-8)	Internalization, signaling	Polyubiquitination	[40]
CXCR4	AIP4	USP14, USP8 (indirectly)	Three Lys in C-term	Agonist (SDF-1α = CXCL12)	Lysosomal degradation; together with STAM-1 role in p44/42 MAPK activation	β-arrestin 1 involved; DTX3L–controls sorting to lysosomes by blocking activity of AIP4	[41,42,43,44,45,46,47,48]
CXCR7	N.D.	Upon stimulation	Lys in C-term	Constitutive	Ubiquitination required for membrane expression of the receptor	β-arrestin involved	[49]
Class A Orphans							
GPR37	Parkin	N.D.	C-term	Constitutive	ERAD	N.D.	[50]
	HRD1	N.D.	N.D.	Induced by overexpression of ATF6	ERAD	Degradation of GPR37 reduces ER stress induced apoptosis	[51]
Dopamine Receptors							
D_1_R, D_2_R	N.D.	N.D.	N.D.	Constitutive	N.D.	N.D.	[52]
D_4_R	Cullin3	N.D.	Non-Lys ubiquitination	Constitutive	Does not influence degradation	Polyubiquitination	[28,52,53]
D_5_R	N.D.	N.D.	N.D.	Constitutive	N.D.	N.D.	[52]
Glycoprotein hormone receptors							
FSH	N.D.	N.D.	Mainly in IC3	Constitutive	Cell-surface expression	Other residues can also be ubiquitinated	[54]
Lysophospholipid receptors							
LPA_2_	N.D.	N.D.	N.D.	Agonist (LPA)	Proteasomal degradation, cell survival	N.D.	[55]
S1P_1_	WWP2	N.D.	N.D.	Functional antagonist (FTY720P)	Proteasomal degradation	Polyubiquitination	[56,57]
Melanocortin receptors							
MC_2_	Mahogunin	N.D.	N.D.	Agonist (ACTH)	N.D.	Multi-monoubiquitination	[58]
Opioid receptors							
δ (DOR)	AIP4	N.D.	Lys	Agonist (DADLE)	Proteasomal degradation	Polyubiquitination, stimulates transport to ILVs	[59,60,61,62]
	N.D.	N.D.	N.D.	Select. agonist (Deltropin I)	Lysosomal degradation	Co-degradation with MOR	[63]
	N.D.	N.D.	N.D.	Constitutive	Proteasomal degradation	ER-retained receptor	[64]
κ (KOR)	N.D.	N.D.	Lys^338^, Lys ^349^, Lys ^378^ in C-term	Constitutive but enhanced by agonists	Lysosomal and proteasomal degradation	Lys^63^ polyubiquitination; β-arrestin involved; enhanced by receptor phosphorylation	[65,66]
µ (MOR)	N.D.	N.D.	Residue in IC1	Agonist (DAMGO, DADLE)	Lysosomal and proteasomal degradation	β-arrestin 1 involved	[67,68,69]
	Smurf2	N.D.	Lys^94^ and Lys^96^ in IC1	Non-selective agonist (DADLE)	Internalization by controlling maturation of the receptor-containing CCPs	Polyubiquitination; β-arrestin 2 involved	[59]
	N.D.	N.D.	N.D.	DOR activation (Deltropin)	Co-degradation with DOR in lysosomes	N.D.	[63]
Orexin receptors							
OX_2_	cIAP-1 and -2 are important	N.D.	N.D.	TNF-α	Degradation	N.D.	[70]
P2Y receptors							
P2Y_1_	Nedd4-2	N.D.	Lys in C-term	Agonist (ADP)	p38 MAPK activation	N.D.	[71]
Platelet-activating receptors							
PAF receptor	Cbl is important	N.D.	N.D.	Constitutive	Agonist (PAF)-dependent down-regulation in proteasome and lysosome	Monoubiquitination	[72]
Prostanoid receptors							
IP	N.D.	N.D.	N.D.	Agonist (cicaprost-mature receptor)	Lysosomal degradation of mature receptor; proteasomal degradation of immature receptor	Polyubiquitination	[73]
Proteinase-activated receptors							
PAR1	N.D.	Upon stimulation	Lys^421^, Lys^422^ in C-term	Constitutive and agonist-induced (SFLLRN-NH_2_)	Basal ubiquitination blocks constitutive internalization; agonist-dependent ubiquitination is involved in internalization	N.D.	[74,75]
	Nedd4-2		Lys	Agonist (α-thrombin)	p38 MAPK activation	Lys^63^-type polyubiquitination	[71]
PAR2	Cbl	AMSH and USP8	Lys	Agonist (peptide SLIGR-NH_2_)	Lysosomal degradation	Monoubiquitination; DUBs are essential for lysosomal trafficking	[76,77]
Tachykinin receptors							
NK_1_	N.D.	N.D.	Lys	Agonist (Substance P)	Down-regulation and degradation	N.D.	[78]
Thyrotropin-releasing hormone receptor							
TRH_1_	N.D.	N.D.	N.D.	Constitutive	ERAD	N.D.	[79]
Vasopressin and oxytocin receptors							
V_2_	N.D.	N.D.	Lys^268^ in IC3	Agonist (Arg-vasopr.)	Degradation	β-arrestin 2 involved	[80]
Class B GPCRs							
Glucagon receptors							
GIP	N.D.	N.D.	N.D.	Agonist (GIP)	Proteasomal degradation	N.D.	[81]
Parathyroid hormone receptors							
PTH1	N.D.	USP2	N.D.	Activating PTH [1,2,3,4,5,6,7,8,9,10,11,12,13,14,15,16,17,18,19,20,21,22,23,24,25,26,27,28,29,30,31,32,33,34] and non-activating PTH [7,8,9,10,11,12,13,14,15,16,17,18,19,20,21,22,23,24,25,26,27,28,29,30,31,32,33,34] ligands	PTH [7,8,9,10,11,12,13,14,15,16,17,18,19,20,21,22,23,24,25,26,27,28,29,30,31,32,33,34]-induced proteasomal degradation	Lys^48^-type polyubiquitination	[82]
Class C GPCRs							
Calcium-sensing receptors							
CaS	Dorphin	N.D.	Lys	Constitutive	ERAD	N.D.	[83]
GABA_B_ receptors							
GABA_B1_	N.D.	USP14	Lys	Constitutive and induced by PMA	Internalization and lysosomal degradation	N.D.	[84]
Metabotropic glutamate receptors							
mGlu_1a_ mGlu_5_	Siah1A	N.D.	Lys	Constitutive	Proteasomal degradation	N.D.	[85]
Class Frizzled GPCRs							
FZD_4_	N.D.	USP8	N.D.	Constitutive	Internalization; lysosomal degradation	N.D.	[86]

N.D., non-defined; A_2A_, Adenosine A_2A_ receptor; Nedd4, Neural precursor cell-expressed developmentally downregulated gene 4; IC1 or 3, Intracellular loop 1 or 3; MARCH2, Membrane associated ring-CH-type finger 2; AT1, Angiotensin-II receptor type 1; D_1_R-D_5_R, Dopamine D1-D5 receptor; CXCR2-CXCR7, C-X-C chemokine receptor-2- 7; IL-8, Interleukine 8; AIP4, Atrophin-1-interacting protein 4; USP, Ubiquitin-specific protease; CXCL12, C-X-C motif chemokine ligand 12; STAM-1, Signal transducing adapter molecule 1; MAPK; Mitogen-activated protein kinase; DTX3L, Deltex E3 ubiquitin Ligase 3L; HRD1, ERAD-associated E3 ubiquitin-protein ligase HRD1; ATF6, Activating transcription factor 6; ERAD, Endoplasmic reticulum-associated degradation; ER, Endoplasmic reticulum; FSH, Follicle-stimulating hormone receptor; LPA_2_, Lysophosphatidic acid receptor 2; S1P1, Sphingosine-1-phosphate receptor 1; WWP2, WW domain-containing protein 2; ACTH, Adrenocorticotropic hormone; DOR, δ-opioid receptor; KOR, κ-opioid receptor; MOR, µ-opioid receptor; ILV, Intraluminal vesicle; DADLE, [D-Ala2, D-Leu5]-Enkephalin; DAMGO, [d-Ala2, N-MePhe4, Gly-ol]-enkephalin; Smurf2, SMAD specific E3 ubiquitin protein ligase 2; CCPs, Clathrin-coated pits; OX_2_, Orexin receptor 2; cIAP-1, Cellular inhibitor of apoptosis protein-1; TNF-α, Tumor necrosis factor α; P2Y_1_, P2Y purinoceptor 1; PAF, Platelet-activating factor receptor; IP, Prostanoid IP receptor; PAR1/PAR2, Proteinase-activated receptor 1 and 2; Cbl, E3 ubiquitin-protein ligase Cbl; AMSH, Associated molecule with the SH3-domain of STAM; NK_1_, Neurokinin-1 receptor; TRH1, Thyrotropin-releasing hormone receptor; GIP, Gastric inhibitory polypeptide; PTH1, Parathyroid hormone 1 receptor; CaS, Calcium-sensing receptor 1; GABA_B1_, Gamma-aminobutyric acid B1; PMA, phorbol 12-myristate 13-acetate; mGlu_1a_/mGlu_5,_ Metabotropic glutamate receptor 1a or 5; Siah1A, Seven in absentia 1A; FZD4, Frizzled-4 receptor.
